# Targeting the Aryl Hydrocarbon Receptor With Indole-3-Aldehyde Protects From Vulvovaginal Candidiasis via the IL-22-IL-18 Cross-Talk

**DOI:** 10.3389/fimmu.2019.02364

**Published:** 2019-10-11

**Authors:** Monica Borghi, Marilena Pariano, Valentina Solito, Matteo Puccetti, Marina M. Bellet, Claudia Stincardini, Giorgia Renga, Carmine Vacca, Federica Sellitto, Paolo Mosci, Stefano Brancorsini, Luigina Romani, Claudio Costantini

**Affiliations:** ^1^Department of Experimental Medicine, University of Perugia, Perugia, Italy; ^2^Department of Pharmaceutical Sciences, University of Perugia, Perugia, Italy; ^3^Department of Veterinary Medicine, University of Perugia, Perugia, Italy

**Keywords:** AhR, IL-22, IL-18, vulvovaginal candidiasis, 3-IAld

## Abstract

Vulvovaginal candidiasis (VVC) is a common mucosal infection caused by *Candida* spp., most frequently by *Candida albicans*, which may become recurrent and severely impacting the quality of life of susceptible women. Although it is increasingly being recognized that mucosal damage is mediated by an exaggerated inflammatory response, current therapeutic approaches are only based on antifungals that may relieve the symptomatology, but fail to definitely prevent recurrences. The unrestrained activation of the NLRP3 inflammasome with continuous production of IL-1β and recruitment of neutrophils is recognized as a pathogenic factor in VVC. We have previously shown that IL-22 is required to dampen pathogenic inflammasome activation in VVC via the NLRC4/IL-1Ra axis. However, IL-22 also regulates IL-18, a product of the inflammasome activity that regulates IL-22 expression. Here we describe a cross-regulatory circuit between IL-18 and IL-22 in murine VVC that is therapeutically druggable. We found that IL-18 production was dependent on IL-22 and NLRC4, and that IL-18, in turn, contributes to IL-22 activity. Like in IL-22 deficiency, IL-18 deficiency was associated with an increased susceptibility to VVC and unbalanced Th17/Treg response, suggesting that IL-18 can regulate both the innate and the adaptive responses to the fungus. Administration of the microbial metabolite indole-3-aldehyde, known to stimulate the production of IL-22 via the aryl hydrocarbon receptor (AhR), promoted IL-18 expression and protection against *Candida* infection. Should low levels of IL-18 be demonstrated in the vaginal fluids of women with recurrent VVC, targeting the AhR/IL-22/IL-18 pathway could be exploited for future therapeutic approaches in VVC. This study suggests that a deeper understanding of the mechanisms regulating inflammasome activity may lead to the identification of novel targets for intervention in VVC.

## Introduction

*Candida albicans* is a human commensal of the skin, gastrointestinal tract and vagina that causes pathologies, collectively known as candidiasis, upon abnormal colonization (1). Clinical manifestations of candidiasis range from cutaneous and mucosal infections to invasive candidiasis with bloodstream and deep-seated infections (1), with distinct prevalence (2), and pathogenesis (3). A common mucosal infection of otherwise healthy women is vulvovaginal candidiasis (VVC). It is suggested that about 75% of women have experienced at least one episode during their lifetime (4) while recent estimates indicate that 138 million women annually suffer from recurrent VVC, a debilitating condition with four or more episodes within a year that severely affect the quality of life (4). Current therapy for recurrent VVC involves induction regimen of antifungals followed by a maintenance therapy for at least 6 months. However, upon antifungal cessation, about 50% of patients experience a relapse and no definite cure can be guaranteed (5).

While current therapeutic approaches in recurrent VVC target the fungus to relieve the symptomatology, it is becoming increasingly clear that the mucosal damage is propagated by the host immune response, which is characterized by an exaggerated inflammatory state (3). Current models on VVC etiopathogenesis suggest that epithelial cell activation by *Candida* hyphal transition results in the production of inflammatory mediators, including S100A8 and IL-1β by NLRP3 inflammasome, and recruitment of polymorphonuclear cells (PMN) that are however ineffective in reducing fungal burden, likely because of a functional anergy induced by heparan sulfate (6). The fungus can therefore overgrow and initiate an amplifying cascade of epithelial cell activation, neutrophil recruitment and production of inflammatory mediators, that results in chronic inflammation and tissue damage (6). That NLRP3 inflammasome is a crucial molecular mechanism contributing to host immunopathology in VVC has recently been confirmed using comprehensive genomic, immunological, and pharmacological approaches (7). Thus, interfering with this pathological cascade might represent a therapeutic opportunity to limit the inflammatory response and prevent tissue damage. We have recently shown that, in the physiological response to *Candida* colonization in the vagina, the activity of the NLRP3 inflammasome is strictly regulated (8). Indeed, the cytokine IL-22, produced in response to the activation of the aryl hydrocarbon receptor (AhR) (9), promoted the activation of NLRC4 in epithelial cells, with subsequent production of IL-1 receptor antagonist (IL-1Ra) that in turn restrained NLRP3 activity and IL-1β production (8). IL-1Ra was defective in symptomatic infection in mice and humans and administration of the recombinant IL-1Ra anakinra could prevent the pathogenic inflammasome activity (8). Although IL-1β was clearly associated with immunopathology in VVC, our study showed that additional inflammasome-dependent cytokines, such IL-1α and IL-18, were produced during infection (8). While the pattern of production and the role of IL-1α appeared to mirror that of IL-1β, IL-18 showed a bimodal peak production, being expressed both in the early and the late phases of the infection; moreover, neutralization of IL-18 resulted in worsened vaginal pathology and increased levels of myeloperoxidase, S100A8 and S100A9 (8). These results suggest that IL-18, at variance with IL-1 cytokines, might play a protective role during infection in VVC, although the mechanisms regulating its production and mediating its functions are unexplored. Interestingly, IL-22 and IL-18 are emerging as strictly intertwined cytokines that cross-regulate each other expression to modulate the immune response. Indeed, Munoz *et al*. have shown that IL-22 increased IL-18 expression in intestinal epithelial cells during *T. gondii* and *C. rodentium* infection and, conversely, IL-18 was required for the expression of IL-22 in innate lymphoid cells after *T. gondii* infection (10). Similarly, IL-18, in combination with IL-15, induced the proliferation of human ILC3s and promoted the expression of IL-22 by activating the NF-κB component p65 and its binding to the *Il22* promoter (11).

Based on these premises, in the present study we evaluated whether a cross-talk between IL-22 and IL-18 was effective in a murine model of VVC. We found that the production of IL-18, initially mediated by NLRP6, was later sustained by the IL-22/NLRC4 axis. IL-18, in turn, increased the levels of bioactive IL-22 and the activation of NLRC4 in a positive feedforward loop. Triggering IL-22 production via the AhR agonist indole-3-aldehyde (3-IAld), a metabolite derived from the microbial degradation of tryptophan and present in the vaginal fluid of mice (9), promoted the expression of IL-18 and the resolution of inflammation in VVC. These results suggest that the AhR/IL-22/IL-18 pathway could represent a druggable target in VVC.

## Materials and Methods

### Mice

Female C57BL/6, 8–10 weeks of age, and *B6.129P2-Il18tm1Aki/J*, 4–8 weeks of age, were purchased from Charles River (Calco, Italy). *Ahr*^−/−^ mice were kindly provided by Prof. Francesca Fallarino (University of Perugia). Homozygous *Nlrp3*^−/−^, *Nlrc4*^−/−^, and *Il22*^−/−^ mice on the C57BL/6 background were bred under specific pathogen-free conditions at the Animal Facility of the University of Perugia, Perugia, Italy.

### Vaginal Infection and Treatment

Mice were injected subcutaneously (sc) with 100 μl of 1 g/ml β-estradiol 17–valerate (Sigma Chemical Co.) dissolved in sesame oil (Sigma-Aldrich) 48 h before vaginal infection. Estrogen administration continued weekly until completion of the study to maintain pseudoestrus. The estrogen-treated mice were inoculated intravaginally with 20 μl of a phosphate-buffered saline (PBS) suspension of 5 × 10^6^ viable *C. albicans* blastospores from early–stationary-phase cultures (i.e., 18 h of culture at 36°C in Sabouraud-dextrose agar with chloramphenicol plates, BD Diagnostics). The time course of infection was monitored in individual mice by culturing 100 μl of serially diluted (1:10) vaginal lavages on Sabouraud-dextrose agar with chloramphenicol plates. Vaginal lavages were conducted using 100 μl of sterile PBS with repeated aspiration and agitation. CFUs were enumerated after incubation at 37°C for 24 h and expressed as log10 CFU/100 μl of lavage fluid. In addition, PMN enumeration was accomplished by cytospin preparations of the vaginal fluids (VF), which were stained with May-Grünwald-Giemsa and observed through a BX51 microscope equipped with a high-resolution DP71 camera (Olympus). PMN were identified by nuclear morphology and enumerated per field at × 100 magnification. 3-IAld (Sigma Chemical Co.) was dissolved in DMSO, diluted with either PBS (for the intragastric-ig-administration) or sesame oil (sc and intraperitoneal-ip-administration), and administered ip, ig, or sc every day starting the day of infection at the dose of 18 mg/kg, as previously shown (9). None, mice receiving sesam oil sc together with estrogen for the infection.

### SiRNA Design and Delivery

Predesigned SiRNA against murine NLRP6 (MMC.RNAI.N001081389.12.1) were purchased from Integrated DNA Technologies (IDT, TEMA Ricerca). Each mouse was intravaginally treated with unmodified SiRNA (0.2 μg) or equivalent doses of non-specific control SiRNA duplex in a volume of 20 μl of duplex buffer (IDT, TEMA Ricerca). Intravaginal SiRNA was given once the day before infection and daily after infection.

### Histological Analysis

For histology, the entire vaginas were removed and immediately fixed in 10% neutral buffered formalin (Bio-optica) for 24 h. The fixed organs were dehydrated, embedded in paraffin, sectioned into 3–4 μm and stained with periodic acid-Schiff reagent. Histology sections were observed and acquired using a microscope (BX51 Olympus) with a 40× objective and the analySIS image processing software (Olympus).

### Immunofluorescence and Immunohistochemical Staining

The vagina was removed and fixed in 10% phosphate-buffered formalin, embedded in paraffin and sectioned at 5 μm. Then, vagina sections were stained with anti-NALP6 (F-20) (sc-50636, Santa Cruz) followed by anti-goat TRITC (Biolegend), anti-NLRP3 (ab4207, Abcam) antibody followed by anti-goat TRITC (Biolegend), anti-phosphorylated NLRC 4 (Nlrc4-P) (Genentech) followed by anti-hamster FITC (Sigma), anti-IL-22 (PA-21356 Invitrogen), anti-IL22ra2 (SC-134975, Santa Cruz) or anti-AhR (SC-101104, Santa Cruz) antibody. All mAbs were incubated overnight at 4°C. 4′-6-Diamino-2-phenylindole (DAPI, Molecular Probes) was used to counterstain tissues and to detect nuclei. For immunohistochemistry, the sections were incubated overnight with polyclonal anti-IL-18 (ab71495, Abcam) at dilution of 1:200 followed by the secondary biotinylated antibody. Tissues were counterstained with hematoxylin. All the images were acquired using a microscope (BX51 Olympus) with a 40× objective and the analySIS image processing software (Olympus).

### ELISA and Real-Time PCR

The levels of murine cytokines in the VF were determined by ELISA kits (eBioscience and R&D System) following manufacturer's instructions. Data were normalized to total protein levels for each sample as determined using the Bio-Rad Protein assay (Life Science, Bio-Rad Laboratories S.r.l.) and results represent mean cytokine levels (± s.e.m.) from samples pooled from three similar experiments (*n* = 3–4 total samples per group). Real-time PCR was performed using the iCycler iQ detection system (Bio-Rad) and iTaq™ Universal SYBR® Green Supermix (Biorad). Organs were lysed and total RNA was extracted using RNeasy Mini Kit (QIAGEN) and was reverse transcribed with Sensiscript Reverse Transcriptase (QIAGEN) according to the manufacturer's directions. Amplification efficiencies were validated and normalized against β-actin. The thermal profile for SYBR Green real-time PCR was at 95°C for 3 min, followed by 40 cycles of denaturation for 30 s at 95°C and an annealing/extension step of 30 s at 60°C. Each data point was examined for integrity by analysis of the amplification plot. The mRNA-normalized data were expressed as relative mRNA in knockout vs. wild-type mice and infected vs. naïve mice.

### Statistical Analysis

Student's *t*-test, one- or two-way ANOVA with Bonferroni *post-hoc* test were used to determine the statistical significance. Significance was defined as *p* < 0.05. Data are pooled results (mean ± SEM) or representative images from three experiments. GraphPad Prism software 6.01 (GraphPad Software) was used for analysis.

## Results

### IL-18 Production in VVC Is Dependent on the Sequential Activation of NLRP6 and NLRC4

**Figure 1:**
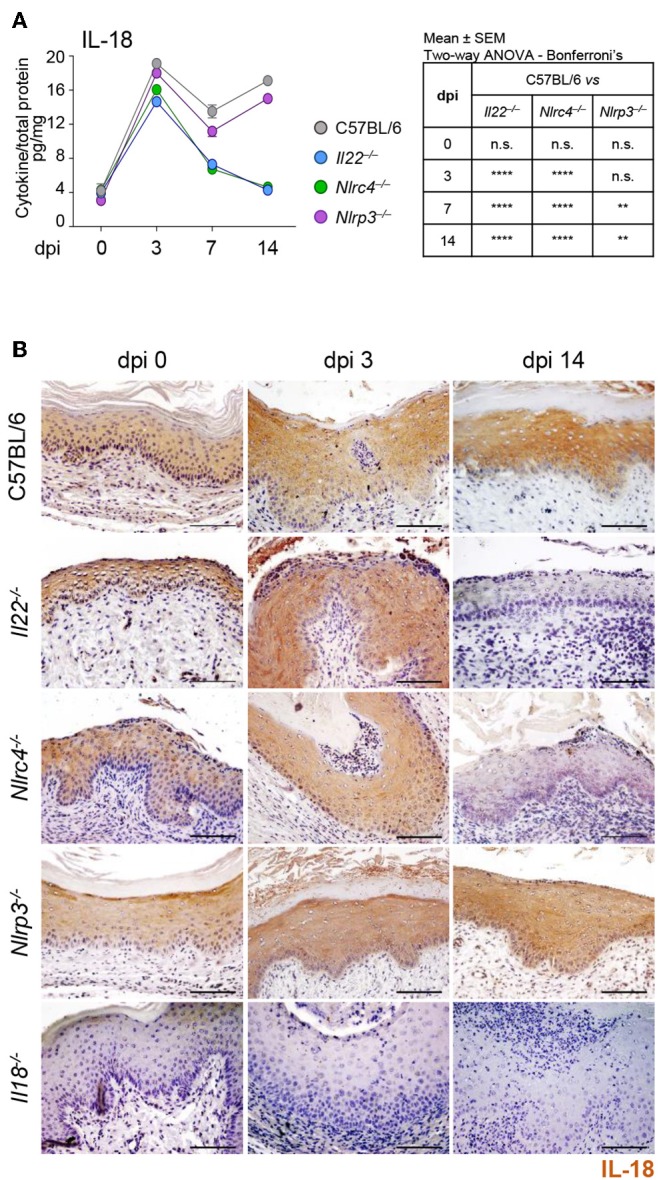
IL-18 production in VVC depends on the IL-22/NLRC4 axis. C57BL/6, *Il22*^−/−^*, Nlrc4*^−/−^, *Nlrp3*^−/−^, and *Il18*^−/−^ mice (*n* = 6 mice/group) were intravaginally inoculated with 5 × 10^6^ *C. albicans* blastoconidia and assessed for **(A)** IL-18 production (pg/mg, cytokine/total proteins for each sample) in vaginal fluids at different days post-infection (dpi). Results represent mean cytokine levels from samples pooled from four experiments (*n* = 3–4 total samples per group). **(B)** Immunohistochemistry of vaginal sections with antibody to IL-18 (Scale bars, 100 μm). Images were acquired with a 40× objective and the analySIS image processing software. Hematoxylin-Eosin staining was used to counterstain tissues. *Il18*^−/−^ mice were used as negative staining control. **P < 0.01 and ****P < 0.0001 knockout vs. wild-type mice at different dpi (see table in **A**).

To assess whether a cross-talk between IL-22 and IL-18 takes place in murine VVC, we resorted to C57BL/6 mice intravaginally infected with *Candida* blastospores and first evaluated the levels of IL-18 in VF and the cellular sources. In agreement with previous findings (8), IL-18 showed a bimodal production, with a first peak in the initial phase of infection and a second one in a later phase (Figure 1A). Immunohistochemical analysis demonstrated a primary localization of IL-18 in epithelial cells (Figure 1B), likely representing the main producers of IL-18 in VVC. To assess whether IL-18 production was dependent on IL-22, *Il22*^−/−^ mice were infected with *Candida* blastospores and IL-18 production was evaluated by ELISA and immunohistochemistry. As shown in Figures 1A,B, the peak of IL-18 production in the initial phase of infection was not affected in mice deficient of IL-22. IL-22 was instead required for the production of IL-18 late in infection, as the peak production at 14 days post-infection was abrogated in *Il22*^−/−^ mice. Interestingly, *Nlrc4*^−/−^ mice phenocopied *Il22*^−/−^ mice as IL-18 levels were unaffected in the early phase of the infection but dramatically reduced in the later phase (Figures 1A,B), consistent with previous findings showing that IL-22 and NLRC4 works along the same pathway (8).

**Figure 2:**
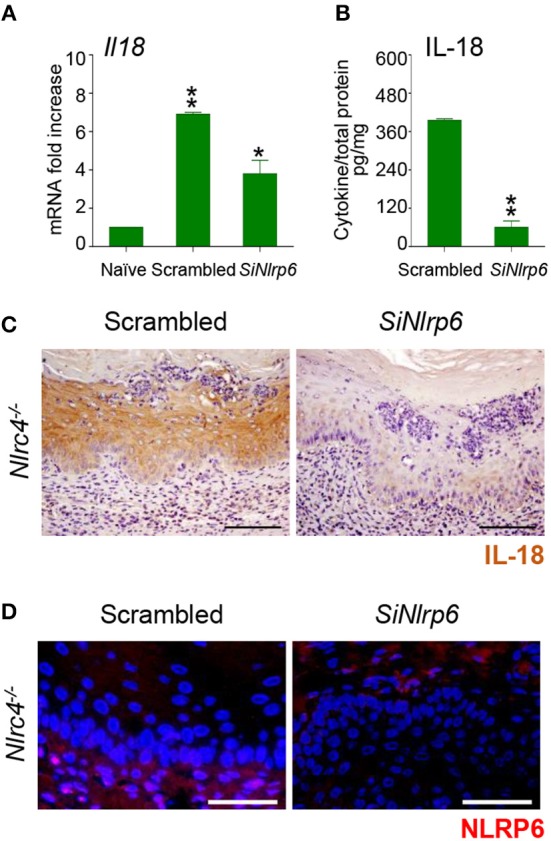
NLRP6 drives early IL-18 production in VVC. **(A)** *Il18* gene expression (RT-PCR) in vaginal tissues and **(B)** IL-18 protein production (pg/mg, cytokine/total proteins for each sample) in vaginal fluids at 3 dpi in *Nlrc4*^−/−^ (*n* = 6) mice intravaginally infected and treated with SiRNA against NLRP6 or equivalent doses of non-specific control SiRNA (scrambled) duplex. *P < 0.05, **P < 0.01 SiNLRP6 vs. scrambled. **(C)** Immunohistochemistry of vaginal sections with antibody to IL-18. Hematoxylin-eosin staining was used to counterstain tissues. **(D)** Immunofluorescence of vaginal sections with antibody to NLRP6. Images were acquired with a 40× objective and the analySIS image processing software (Scale bars, 50 μm).

To gain insights into the initial production of IL-18, we evaluated the contribution of NLRP3, known to be activated in VVC (8) and able to produce IL-18 (12) as well as of NLRP6, known to produce IL-18 in different experimental settings (13–16). We found a significant reduction of IL-18 levels in *Nlrp3*^−/−^ mice at 7 and 14 dpi but not at 3 dpi (Figures 1A,B). In contrast, on inhibiting NLRP6 on *Nlrc4*^−/−^ mice by specific SiRNA – that efficiently down-regulated NLRP6 expression (Figure 2D and Supplementary Figure 1)—IL-18 mRNA induction in vaginal tissue (Figure 2A), the protein levels in VF (Figure 2B) and the IL-18 staining in epithelial cells (Figure 2C) were all significantly reduced. Collectively, these results indicate that the production of IL-18, while mediated by NLRP6 in the initial phase of infection, is then dependent on NLRC4, more than NLRP3. As IL-22 promotes NLRC4 activity in infection (8), this highlights the cross-talk between IL-22 and IL-18 in VVC.

### IL-18 Promotes IL-22-Dependent Protection in VVC

**Figure 3:**
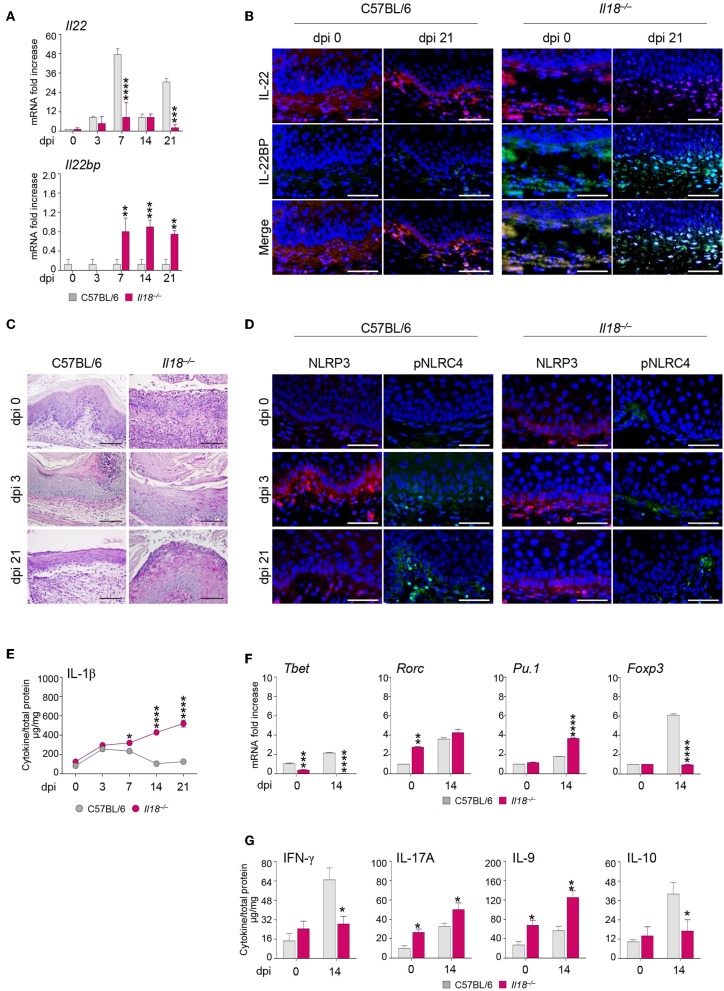
*Il18*^−/−^ mice are susceptible to VVC. *Il18*^−/−^ mice were intravaginally inoculated with 5 × 10^6^ *C. albicans* blastoconidia and were monitored for: *Il22* and *Il22bp* gene **(A)** and protein **(B)** expression in the vagina by RT-PCR and immunofluorescence staining, respectively, at different days post-infection (dpi). **(C)** Vaginal histology (Periodic Acid Shiff–staining of vaginal sections. Scale bars, 100 μm). **(D)** Immunofluorescence staining of vaginas at different dpi with antibodies to NLRP3 or p-NLRC4. Sections were stained with the relevant primary antibody overnight at 4°C followed by the secondary TRITC or FITC antibody. Images were acquired using a fluorescence microscope with a 40× objective (Scale bars, 50 μm) and the analySIS image processing software. 4′-6-Diamino-2-phenylindole was used to counterstain tissues and to detect nuclei. **(E)** IL-1β production (μg/mg, cytokine/total proteins for each sample) in vaginal fluids at different dpi. Results represent mean cytokine levels from samples pooled from four experiments (*n* = 3–4 total samples per group). **(F)** Transcriptional factors gene expression on lumbar lymph node by RT-PCR and **(G)** cytokine levels in the vaginal fluids by ELISA at 14 dpi. *P < 0.05, **P < 0.01 and ***P < 0.001, ****P < 0.0001 knockout vs. wild-type mice.

Further studies in *Il18*^−/−^ mice revealed that IL-18 in turn regulated IL-22 activity in murine VVC. Indeed, we found that IL-22 mRNA (Figure 3A) and protein (Figure 3B) expression was impaired in these mice as opposed to wild-type mice in infection (Figures 3A,B). In contrast, the IL-22 binding protein (IL-22BP) mRNA (Figure 3A) and protein (Figure 3B) expression was greatly induced.

Thus, the relative absence of IL-18 affects IL-22 activity by tipping the balance in favor of the inhibitory molecule IL-22BP (17). Consistently, *Il18*^−/−^ mice showed a severe and long-lasting vaginal immunopathology characterized by the presence of fungal and inflammatory cells infiltrating the vaginal parenchyma with signs of epithelial damage (Figure 3C), a prolonged activation of NLRP3, but not of phospho-(p)NLRC4 (Figure 3D), and a higher production of IL-1β (Figure 3E). These results point to a defective IL-22/NLRC4 activity leading to sustained NLRP3/IL-1β in conditions of IL-18 deficiency. As acquired Th1 (18, 19), Th17 (20), Th9 (21), and regulatory T cell (22) immunity have been described in murine and human VVC, we also have evaluated whether the acquired Th immune response was altered in *Il18*^−/−^ mice with VVC. The levels of the transcription factors characteristic of the different T cell subsets in the draining lymph nodes were indicative of an upregulation of the inflammatory Th17 and Th9 cell subsets, and an impairment of the Th1/Treg cell subsets (Figure 3F), in line with the protein levels of the corresponding cytokines in the vagina (Figure 3G). Collectively, these results would suggest an aberrant Th cell activation in the relative absence of IL-18.

Of note, the phenotype of *Il18*^−/−^ mice strongly resembles that observed in *Il22*^−/−^ mice (8), further pointing to the collaborative IL-18 and IL-22 cross-talk in protection against *Candida* vaginal infection. These results indicate that IL-18 modulates IL-22 activity and regulates both innate and adaptive mechanisms of immune protection, thus representing an important player in the host response to *Candida* infection.

### 3-IAld Protects From VVC by Promoting the IL-22/IL-18 Cross-Talk *via* AhR

**Figure 4:**
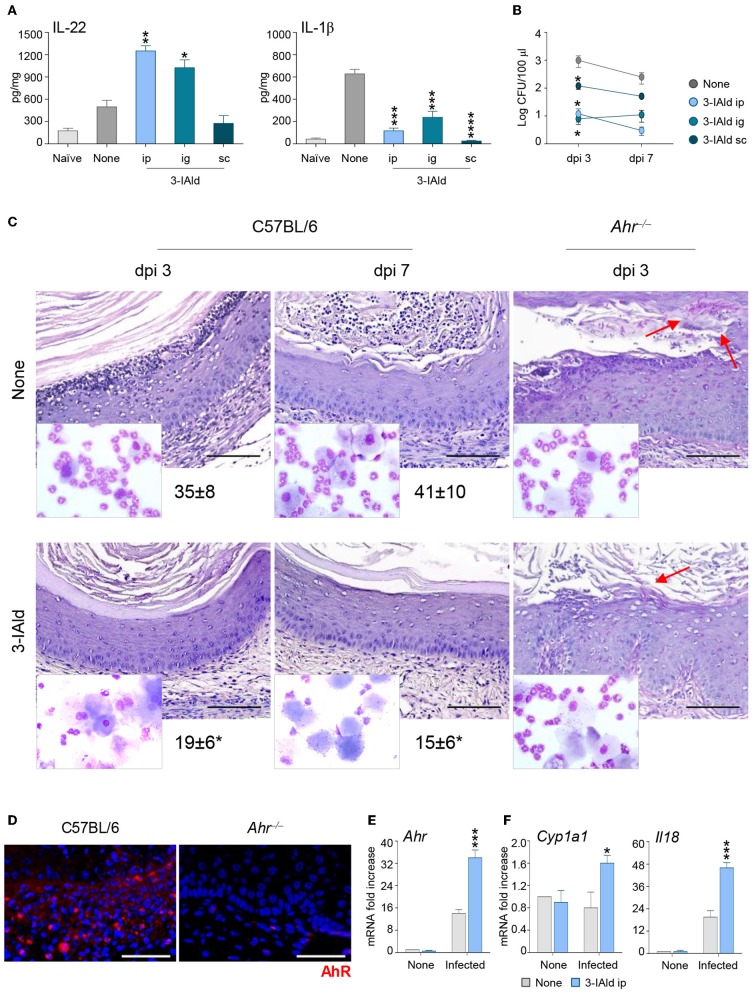
3-IAld promotes resistance to VVC by inducing the IL-22/IL-18 axis via AhR. **(A)** IL-22, IL-1β production (pg/mg, cytokine/total proteins for each sample), and **(B)** fungal growth (mean log CFUs ± s.e.m.) at 7 dpi in vaginal fluids of C57BL/6 mice (*n* = 6 mice/group) inoculated with 5 × 10^6^ *C. albicans* blastoconidia and treated subcutaneously (sc), intraperitoneally (ip), or intragastrically (ig) with 3-IAld every day starting the day of infection. **(C)** C57BL/6 and *Ahr*^−/−^ mice (*n* = 6 mice/group) with VVC were ip treated with 3-IAld every day starting the day of infection and assessed for vaginal histology (Periodic Acid Shiff–staining of vaginal sections (scale bars, 100 μm) and PMN recruitment in the VF (mean % ± s.e.m., in the insets) at different dpi. Images were acquired using a microscope with a 40× objective and the analiSIS image processing software. Red arrows indicate fungi. AhR protein **(D)** and gene **(E)** expression by immunofluorescence staining of vaginal sections (Scale bars, 100 μm) and RT-PCR of vaginal tissues, respectively, in infected mice treated with 3-IAld. Images were acquired with a 40× objective and the analySIS image processing software. **(F)** Gene expression by RT-PCR at 3 (*AhR* and *Cyp1a1*) and 7 (*Il18*) dpi. *P < 0.05, **P < 0.01 and ***P < 0.001, ****P < 0.0001 treated vs. untreated (None) mice.

To evaluate whether the cross-talk between IL-22 and IL-18 could represent a potential therapeutic target in VVC, we treated infected C57BL/6 mice with the microbial product 3-IAld that we have recently identified and characterized as critical in the maintenance of epithelial integrity by binding to AhR on ILC3s and promoting the expression of IL-22 (9). IL-22-producing ILC3s were indeed present in the vagina and expanded in VVC in wild-type mice, but not in IL-22-deficient mice, while IL-22 production was significantly decreased in AhR-deficient mice, these findings clearly demonstrating that IL-22 was produced by vaginal ILC3s via AhR (22). We have already shown that AhR expression was increased during VVC and AhR-deficient mice were more susceptible to the infection (22). These results, together with the finding that 3-IAld was detected in the VF of VVC mice (9), suggest that, similarly to the gastrointestinal tract, 3-IAld may play a role in epithelial barrier function also in the vagina. The administration of 3-IAld, but not vehicles, via different routes was able to promote IL-22 production, decrease that of IL-1β (Figure 4A) and reduce the fungal burden (Figure 4B) although to a variable degree depending on the route of administration. These effects were paralleled by a decreased PMN influx in the vagina and an improved vaginal pathology in C57BL/6, but not *Ahr*^−/−^, mice (Figure 4C), confirming that the activity of 3-IAld is mediated by AhR. In line with this finding, the expression of AhR (Figures 4D,E) and its specific target *Cyp1a1* (Figure 4F) were both increased in C57BL/6 mice treated with 3-IAld. Interestingly, 3-IAld also induced the expression of IL-18 (Figure 4F), thus suggesting that 3-IAld can engage the cross-talk between IL-22 and IL-18 to mediate protection in VVC.

## Discussion

**Figure 5:**
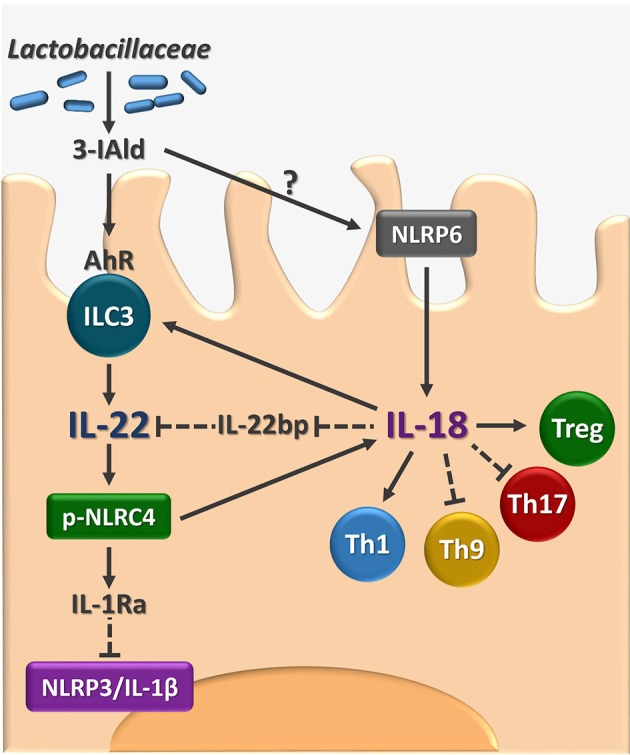
Proposed model for the role of AhR/IL-22/IL-18 axis in VVC. The figure shows that the microbial ligand 3-IAld may activate AhR on vaginal ILC3 to promote IL-22 activity that encompasses the inhibition of the inflammatory NLRP3/IL-1β axis and the production of IL-18 with the contribution of NLRC4 and NLRP6. The IL-22-IL-18 positive cross-talk is apparently required for the generation of innate and adaptive immune protection in VVC. 3-IAld, indole-3-aldheyde. AhR, aryl hydrocarbon receptor. ILC3, type 3 innate lymphoid cells. IL-22BP, IL-22 binding protein; pNLRC4, phospho-NLRC4; Th, T helper cells; Treg, regulatory T cells. Solid lines, activation, dashed lines, inhibition.

The present study identifies IL-18 as an additional player in the host-*Candida* cross-talk in VVC and, by integrating our previous findings (8), delineates a model in which the response to the fungus is orchestrated by the timely activation of different inflammasomes (Figure 5). Specifically, during the initial phase of infection, NLRP3 inflammasome is activated to produce IL-1β that promotes the recruitment of neutrophils and the onset of the inflammatory response. In parallel, NLRP6 inflammasome produces IL-18 that, in turn, increases the expression of IL-22, a key cytokine for the activation of the NLRC4 inflammasome. NLRC4 is a central hub in our model. Indeed, on the one hand, NLRC4 induces IL-1Ra that restrains NLRP3 activation and the production of IL-1β, thus promoting the resolution of the inflammatory response. On the other hand, NLRC4 promotes the expression of IL-18 that, by inducing IL-22 and downregulating IL-22BP expression, increases the levels of bioactive IL-22 and the activation of NLRC4 in a positive feedforward loop. In addition, in line with the ability of IL-18 to increase indoleamine 2,3-dioxygenase activity (23), IL-18 also appears to regulate the adaptive immune response by shifting the balance toward a Th1/Treg cell-mediated activity.

A central tenet of our model is the cross-talk between IL-22 and IL-18 in the proper immune response to the fungus from which it follows that triggering the IL-22/IL-18 pathway may be beneficial in VVC. We have verified this working hypothesis by resorting to the microbial metabolite 3-IAld because it can induce the production of IL-22 by activating AhR and is detectable in murine VF (9). This approach proved to be effective as 3-IAld was able to restrain inflammation and provide protection in VVC via IL-22 and IL-18.

The interest in post-biotics, i.e., metabolic byproducts of probiotics, has grown considerably in the recent years because of multiple advantages. Being naturally-occurring molecules, they are intrinsically associated with high safety and low toxicity. Moreover, microbial metabolites play fundamental roles in mucosal homeostasis, by safeguarding the integrity of the epithelial barrier and promoting an immune tolerant state that allows microbial commensals to thrive while remaining alerted for the presence of pathological insults. Short-chain fatty acid produced by bacterial fermentation, such as butyrate, are prototypical examples of post-biotics able to promote colonic health and protect against inflammation (24) and indole derivatives, such as 3-IAld, are emerging as an important class of microbial molecules with beneficial effects for the host, as demonstrated by our group (9) and others (25, 26), and further supported by the present study. Altogether, these results strengthen the notion that indole derivatives can work at the interface between the pathogen and the host by coopting immune mechanisms of protection and tolerance to aid resolution of inflammation and restoration of tissue homeostasis.

Besides the cross-talk between IL-22 and IL-18, an interesting feature of our model is the initial involvement of NLRP6 in IL-18 production. NLRP6 has recently emerged for its role in gut homeostasis and protection against intestinal colitis and tumorigenesis (27), although more recently NLRP6 also exacerbated gastrointestinal graft-vs-host disease (28). The microbiome appeared to stimulate the protective roles of NLRP6 while the pathogenic activities were microbiome-independent (28). Thus, it is likely that the protective role of NLRP6 during the initial phase of infection in VVC is stimulated by the vaginal microbiome with the production of IL-18. Although an initial evaluation of the microbial communities using the terminal restriction fragment length polymorphisms could not detect different vaginal bacterial communities in women with or without recurrent VVC (29), a subsequent study using NGS techniques revealed diverse microbial community patterns in VVC patients (30). Irrespective of whether the changes in the vaginal microbiome are a cause or a consequence of the inflammatory pathology in recurrent VVC, it is possible that a defective activation of NLRP6 by the microbiome would underpin the lower levels of IL-18 in the vaginal fluids of VVC patients, a hypothesis that deserves further investigation. Interestingly, however, it is worth mentioning that a healthy vaginal microbiome is generally dominated by *Lactobacillus* species (31) and administration of lactobacilli as probiotics has been proposed for the prevention of recurrent VVC. Although current evidence is still limited to support the use of probiotics as an adjuvant therapy for clinical and mycological cure (32), it is worth noting that lactobacilli can produce 3-IAld and the addition of *L. acidophilus* can protect against VVC (9). It comes that the levels of IL-18 and 3-IAld in VF might be used as biomarkers for susceptibility to inflammation and tissue damage in VVC and might gauge the therapeutic administration of probiotics or post-biotics to restore epithelial barrier protection, or even drive changes in dietary habits. Indeed, not only the composition of the vaginal microbiome is expected to respond to dietary changes (33), but *Candida* itself is sensitive to nutrient availability (34), thus suggesting that the diet might be used to shift the balance of the host-*Candida* cross-talk toward a tolerogenic state.

In conclusion, our study suggests that IL-18 participates in the cross-talk between the host and *Candida* in the vaginal mucosa and promotes the resolution of the inflammatory response via IL-22 and NLRC4 activation that, in turn, sustains IL-18 production (Figure 5). Our preliminary observation seem to suggest lower levels of IL-18 in the VF of patients with recurrent VVC as compared to healthy controls (unpublished observations). Should these data be confirmed, this may pave the way for the development of novel therapeutic approaches aimed at counteracting the exaggerated inflammatory response to the fungus that may encompass microbial AhR agonists that trigger the IL-22/IL-18 pathway.

## Data Availability Statement

All datasets generated for this study are included in the manuscript/Supplementary Files.

## Ethics Statement

The study involving human subjects was carried out in accordance with the recommendations of the University Ethics Committee of Perugia (Prot. 2012-028). All subjects gave written informed consent in accordance with the Declaration of Helsinki. Murine experiments were performed according to the Italian Approved Animal Welfare Authorization 360/2015-PR and Legislative decree 26/2014 regarding the animal license obtained by the Italian Ministry of Health lasting for 5 years.

## Author Contributions

MB, MPa, VS, MMB, CS, GR, CV, and FS designed and performed the experiments. PM, MB and MPu collected the clinical data and samples. MB, SB, LR, and CC analyzed the data and wrote the paper.

### Conflict of Interest

The authors declare that the research was conducted in the absence of any commercial or financial relationships that could be construed as a potential conflict of interest.
