# Supplementary material for: Targeting the Aryl Hydrocarbon Receptor With Indole-3-Aldehyde Protects From Vulvovaginal Candidiasis via the IL-22-IL-18 Cross-Talk
Source: Front Immunol. 2019 Oct 11;10:2364. doi: 10.3389/fimmu.2019.02364 (PMC6798081; doi:10.3389/fimmu.2019.02364)
Supplement: Supplementary file 1 [file Data_Sheet_1.docx]

**TARGETING THE ARYL HYDROCARBON RECEPTOR PROTECTS FROM VULVOVAGINAL CANDIDIASIS VIA THE IL-22-IL-18 CROSS-TALK**

Monica Borghi^1*^, Marilena Pariano^1^, Valentina Solito^1^, Matteo Puccetti^2^, Marina M. Bellet^1^, Claudia Stincardini^1^, Giorgia Renga^1^, Carmine Vacca^1^, Federica Sellitto^1^, Paolo Mosci^3^, Stefano Brancorsini^1^, Luigina Romani^1^, and Claudio Costantini^1*^

^1^ Department of Experimental Medicine, University of Perugia, 06132 Perugia, Italy

^2^ Department of Pharmaceutical Sciences, University of Perugia, 06132 Perugia, Italy

^3^ Department of Veterinary Medicine, University of Perugia, 06132 Perugia, Italy

*** Correspondence**

Monica Borghi Claudio Costantini

monicaborghi@live.com costacla76@gmail.com

**Running title:** AhR-IL-22-IL-18 in vulvovaginal candidiasis

**Keywords:** AhR, IL-22, IL-18, vulvovaginal candidiasis, 3-IAld

**SUPPLEMENTARY MATERIAL**

**
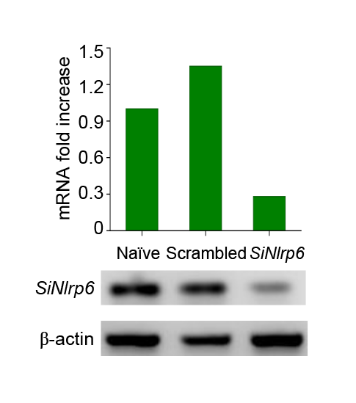
**

**Supplementary Figure 1.** Control of NLRP6 silencing efficiency on infected and uninfected *Nlrc4^–/–^* mice by RT-PCR and electrophoretic separation of amplified fragments. Amplification efficiencies were validated and normalized against β-actin.
